# Efficacy and safety of Lianhua Qingwen in the treatment of patients with moderate COVID-19 infection: A protocol for systematic review and meta analysis

**DOI:** 10.1097/MD.0000000000021614

**Published:** 2020-08-14

**Authors:** Nanyang Liu, Tingting Zhang, Lina Ma, Huican Wang, Yu Cao, Yang Yang, Hui Pei, Hao Li

**Affiliations:** a Xiyuan Hospital, China Academy of Chinese Medical Sciences, Beijing; b College of First Clinical Medicine, Shandong University of Traditional Chinese Medicine, Jinan, Shandong Province, P.R. China.

**Keywords:** coronavirus disease 2019, meta-analysis, pneumonia, systematic review, traditional Chinese herbal medicine

## Abstract

**Background:**

: As of June 2020, more than 7 million cases of coronavirus disease (COVID-2019) have been reported worldwide. At present, there is no vaccine or antiviral for the novel coronavirus pneumonia. Lianhua Qingwen (LQ), a Chinese medicine formula, has been authorized by the Chinese government for treating COVID-2019. This systematic review and meta-analysis will evaluate the efficacy and safety of LQ on patients with COVID-19.

**Methods:**

: Two independent reviewers will search the following databases of the China Biology Medicine disc, China National Knowledge Infrastructure, China Science and Technology Periodical Database, Wanfang database, Embase, PubMed, and Cochrane Library from the date of conception to June 1, 2020. We will use the MeSH/Emtree terms, combining free-text words that were properly adjusted for the different databases in all of the search strategies. We will take primary clinical symptoms, total efficacy, and adverse event into consideration for our primary outcomes. As secondary outcomes, we will estimate the chest computed tomography manifestations, the rate of conversion to severe cases, and secondary clinical symptoms. We will evaluate the quality of including studies through the risk of bias assessment tool provided by the Cochrane Collaboration. Fixed-or random-effect model will be utilized to calculate the overall pooled risk estimates. Forest plots will be generated to prove the pooled results. Sensitivity analysis will be carried out to identify sources of heterogeneity. The Begg rank correlation test and Egger linear regression test will be used to explore publication bias.

**Results:**

: This systematic review and meta-analysis will compare the primary and secondary outcomes at baseline and endpoint in the treatment and control groups to investigate the efficacy and safety of LQ for treatment COVID-2019.

**Discussion:**

Data from this study will provide strong evidence for clinical decision if the findings are positive.

PROSPERO registration number: CRD42020190757.

## 1 Introduction

The severe acute respiratory syndrome coronavirus 2 (SARS-CoV-2), first discovered in December 2019 in Wuhan, China, is a new coronavirus of the same family as SARS-CoV and the Middle East Respiratory Syndrome Coronavirus.^[1]^ The World Health Organization named it coronavirus disease 2019 (COVID-19), which was then declared a pandemic due to widespread infectivity and high infection rate.^[2]^ As of June 2020, it has been estimated that 700,0000 people are suffer from COVID-19 worldwide. Similar to other coronaviruses, general symptoms of SARS-CoV-2 infection are primary flu-like symptoms, such as fever, cough, and fatigue. Severe cases generally present with dyspnea a week after the infection, and some cases rapidly develop into septic shock, acute respiratory distress syndrome, metabolic acidosis that is difficult to correct, and coagulopathy.^[3]^ Currently, there are no registered drugs to treat COVID-19, and vaccine development is not available in the short term. Management is primarily based on supportive therapy and symptomatic treatment to avoid respiratory failure and even death.^[4]^ Several clinical trials of possible COVID-19 treatments are being conducted built on antiviral, anti-inflammatory, immunomodulatory drugs, and other therapies.^[5]^

In the pandemic, the Chinese government serves as a valuable reference in epidemiology, diagnosis, and management worldwide. Chinese herbal medicine, a medical system with local characteristics, was incorporated into management for COVID-19 in the early stage of the onset.^[6,7]^ Lianhua Qingwen (LQ), representative Chinese medicine against respiratory infections caused by viruses, has been used in China for many years.^[8]^ It has proceeded to a large number of clinical and pathological mechanism studies accumulated rich experience. Given the effective virus suppression, LQ has been approved by the China National Health Commission for treating COVID-19.^[9]^

In the preliminary search, we found that despite the increasing number of randomized controlled trials of LQ in the treatment of COVID-19, most clinical trials arise out of low quality and small samples, lacking evidence-based exploration.^[10,11]^ Therefore, we will systematically review the application of LQ in COVID-19 patients to examine the empirical evidence, and provide strong evidence for the clinical practice of COVID-19 pneumonia.

## 2 Methods

The systematic review and meta-analysis has passed the PROSPERO registration (CRD42020168004) on June 11, 2020. We developed the protocol according to the preferred reporting item for systematic review and meta-analysis protocol (PRISMA-P) statement^[12]^ (additional file 1). If there is any amendment, the PROSPERO record will be updated.

### 2.1 Inclusion criteria

#### 2.1.1 Type of participant

Participants with COVID-19 without life-threatening will be included. There will be no restrictions on gender, age, race, or combined with other diseases.

#### 2.1.2 Type of interventions

LQ (capsules, granules, or other types) alone or paired with other routine western medicine will be included. There will be no restrictions regarding the place of origin, dosage form, dosage, and frequency.

#### 2.1.3 Type of comparators

The comparators are likely to include placebo, routine western medicine therapy.

#### 2.1.4 Type of outcome measurements

Our primary outcomes will be the total efficacy, the primary clinical symptoms (fever, cough, fatigue), and the number of patients who had any adverse events at the end of treatment and the end of follow-up. As secondary outcomes, we will estimate the chest computed tomography manifestations, the rate of conversion to severe cases, and the secondary clinical symptoms (expectoration, chest tightness, loss of appetite, and shortness of breath) from baseline to endpoint. If additional outcomes are reported in the eligible study, these results will be extracted and reported.

#### 2.1.5 Type of studies

We will include randomized trials, randomized controlled, or prospective controlled clinical trials. The blind method, sample size, treatment duration, follow-up duration, or publication status will not be limited. English and Chinese publications will be listed.

### 2.2 Exclusion criteria

Exclusion criteria are

1) life-threatening patients with severe pneumonia.
2) Case reports, case series, duplicate reports, letters to editors, comments, and author responses.
3) The full text of the study could not be available.

### 2.3 Databases and search strategy

Relevant trials will be identified in titles/abstracts by 2 independent reviewers search the databases of the China Biology Medicine disc, China National Knowledge Infrastructure, China Science and Technology Periodical Database, Wanfang Database, PubMed, Embase, and Cochrane Library from the date of conception to June 1, 2020. The search terms are: “Chinese medicine”, “traditional Chinese medicine”, “proprietary Chinese medicine”, “Chinese herbal medicine”, “Lianhua Qingwen”, “Lianhua Qingwen”, “novel coronavirus infected pneumonia”, “COVID-19”, “corona virus disease 2019”, “NCP”, “2019-nCOV”, “randomized controlled trial”, “controlled clinical trial”, “randomized”, “randomly”, “trial”. The search words in the Chinese databases are translations of the above words. References from the latest reviews will be searched in case of missing potentially eligible clinical trials.

### 2.4 Data collection and analysis

#### 2.4.1 Study selection

**Figure 1:**
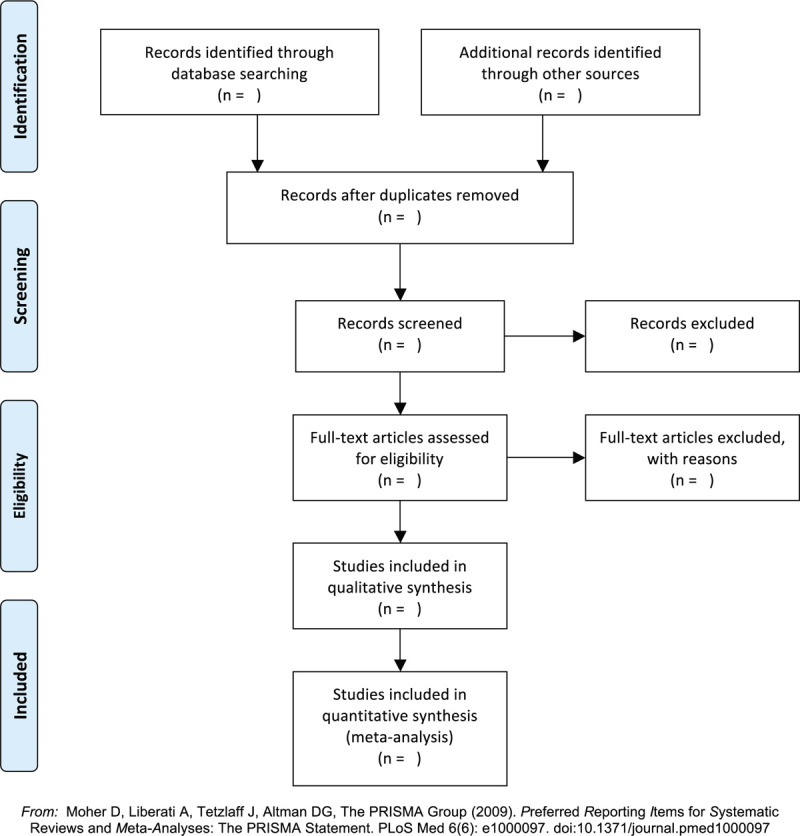
The PRISMA flow chart.

We will export the retrieved records in the database into EndNote X9 software to detect duplicate studies. After removing duplicates, 2 reviewers will independently examine them through read the title and abstract according to the eligibility criteria. If a study potentially eligible, the full text will be obtained and independently reviewed by 2 reviewers. As for the literature that cannot be borne out, it will be confirmed by the discussion of the 2 reviewers. A third reviewer will assist if they are unable to reach an agreement. The PRISMA flow chart is shown in Figure 1.

#### 2.4.2 Data extraction

To ensure the completeness and consistency of the data, 2 independent reviewers will use a pre-designed template to extract data from the eligible studies. The template includes the following items:

(1) general information: first author, corresponding author, contact information,journal, year of publication, country/region, funding source, research design;
(2) characteristics of participants: age, gender, race, education level, disease stage, and severity;
(3) characteristics of study: sample size, random sequence generation, allocation concealment, blinding, follow-up duration;
(4) intervention characteristics: Lianhua Qingwen in the treatment group (dosage form, dose, frequency, duration). Placebo, routine western medicine therapy, and other therapeutic methods in the comparators (drugs, dose, frequency, duration);
(5) Outcomes: the primary outcomes include total efficiency, primary symptoms, and adverse events. Secondary outcomes include the chest computed tomography manifestations, the rate of conversion to severe cases, and secondary clinical symptoms (expectoration, chest tightness, loss of appetite, and shortness of breath). The original author will be contacted if the data is incomplete. That information will be cross-checked by 2 reviewers. Any differences will be discussed and resolved with the third reviewer.

#### 2.4.3 Assessment of the risk of bias

We will assess the quality of included studies through the risk of bias assessment tool provided by the Cochrane collaboration.^[13,14]^ The following items are

(1) random sequence generation and allocation concealment;
(2) blinding of participants, personnel and outcome assessors;
(3) incomplete outcome data;
(4) selective outcome reporting;
(5) and other bias. The risk grade will be judged as low risk of bias, unclear risk of bias, and high risk of bias.

#### 2.4.4 Statistical analysis

Statistical analyses will be conducted using the Review Manager software (version 5.3.5) to calculate the odds ratio, and 95% confidence interval of dichotomous variables. Standardized mean differences or mean differences with 95% confidence interval will be used for the continuous variables. The Mantel–Haenszel method will be utilized for dichotomous variables, while the DerSimonian and Laird inverse variance method will be employed to continuous variables. Heterogeneity between the included studies will be assessed by heterogeneity *χ*2 test and *I*^2^ index. A rough guide to interpretation is as follows: 0% to 40% representing mild heterogeneity; 30% to 60% representing moderate heterogeneity; 50% to 90% representing substantial heterogeneity, and 75% to 100% representing considerable heterogeneity. When heterogeneity cannot be explained, 1 method of analysis is to pool it into a random-effects model to display the results. Otherwise, a fixed-effect model will be used. If quantitative synthesis is not appropriate, we will describe the type of summary planned.

#### 2.4.5 Subgroup analysis

If sufficient studies are determined, we will perform subgroup analysis on the following variables: country/region, sample size, study type, and interventions. Moreover, we will consider further subgroups analysis in the study.

#### 2.4.6 Sensitivity analysis

We will conduct a sensitivity analysis to test the robustness of the pooled results. Furthermore, individual study will be excluded one by one to observe the effect on the pooled results.

#### 2.4.7 Publication bias

If sufficient studies are identified, the Begg rank correlation test or Egger linear regression test will be performed to quantize the publication bias.

#### 2.4.8 Quality of evidence

Two independent reviewers will use the grading of recommendations assessment, development, and evaluation system to estimate the quality of evidence for each result.^[15]^ Each result will be evaluated according to the following five aspects: limitations, inconsistency, indirectness, inaccuracy, and publication bias. The grade will be defined as high, moderate, low, or very low.

## 3 Discussion

It has spread to more than 180 countries/regions around the world since the outbreak of COVID-19 in Wuhan, China, on December 2019, causing significant harm to human health and the social economy.^[16]^ COVID-19 mainly invades the respiratory system, as well as the liver, hilar lymph nodes, heart and blood vessels, and other organs throughout the body.^[17]^ LQ, a prescription composed of a variety of Chinese herbal medicines, has been marketed for more than 10 years since the outbreak of severe acute respiratory syndrome in 2003 in China. Previous studies confirmed that LQ could inhibit influenza virus proteases via multiple targets, making the virus unable to accomplish biotransformation function.^[18]^ In vitro, LQ significantly inhibited the activity of SARS-CoV-2, reduced the virus content in the cell membrane and cytoplasm, and decreased the excessive activation of cytokines.^[19]^ The S protein RBD domain of SARS-CoV-2 supports robust interaction with human (ACE2) molecules and poses a risk to human disease transmission through the binding pathway with S-protein-ACE2.^[20,21]^ Studies demonstrated that honeysuckle and forsythia, the main herbs of LH, can block the binding of multiple ACE2 to S protein to play vital roles in the new coronavirus pneumonia. Recent clinical investigations indicated that LQ capsule significantly improves several clinical symptoms (i.e., fever, cough, fatigue) and shortens the course of COVID-19.^[22,23]^ Because of the benefits, the LQ capsule has been approved by the China National Health Commission for the treatment of COVID-19.^[9]^

As far as we know, this is the first systematic review to examine the empirical evidence of LQ in the treatment of COVID-19. We will evaluate the strengths and limitations based on the existing evidence. This study will be guided by the PRISMA statement to obtain the highest possible quality in the report and methodology. We hope the results of this study could provide a reference for the treatment of traditional Chinese medicine in COVID-19.

## Author contributions

**Conceptualization:** Nanyang Liu, Hao Li.

**Data curation:** Lina Ma, Tingting Zhang.

**Investigation:** Huican Wang, Yu Cao.

**Methodology:** Yang Yang, Hui Pei.

**Supervision:** Hui Pei, Hao Li.

**Validation:** Lina Ma, Hao Li.

**Visualization:** Nanyang Liu, Lina Ma.

**Writing – original draft:** Nanyang Liu, Tingting Zhang.

**Writing – review & editing:** Yu Cao, Hao Li.
